# Kidney tumors associated with germline mutations of *FH* and *SDHB* show a CpG island methylator phenotype (CIMP)

**DOI:** 10.1371/journal.pone.0278108

**Published:** 2022-12-01

**Authors:** Christopher J. Ricketts, J. Keith Killian, Cathy D. Vocke, Yonghong Wang, Maria J. Merino, Paul S. Meltzer, W. Marston Linehan

**Affiliations:** 1 Urologic Oncology Branch, Center for Cancer Research, National Cancer Institute, National Institutes of Health, Bethesda, MD, United States of America; 2 Genetics Branch, Center for Cancer Research, National Cancer Institute, National Institutes of Health, Bethesda, MD, United States of America; 3 Laboratory of Pathology, National Cancer Institute, National Institutes of Health, Bethesda, MD, United States of America; King Faisal Specialist Hospital and Research Center, SAUDI ARABIA

## Abstract

Germline mutations within the Krebs cycle enzyme genes fumarate hydratase (*FH*) or succinate dehydrogenase (*SDHB*, *SDHC*, *SDHD*) are associated with an increased risk of aggressive and early metastasizing variants of renal cell carcinoma (RCC). These RCCs express significantly increased levels of intracellular fumarate or succinate that inhibit 2-oxoglutarate-dependent dioxygenases, such as the TET enzymes that regulate DNA methylation. This study evaluated the genome-wide methylation profiles of 34 RCCs from patients with RCC susceptibility syndromes and 11 associated normal samples using the Illumina HumanMethylation450 BeadChip. All the HLRCC (*FH* mutated) and SDHB-RCC (*SDHB* mutated) tumors demonstrated a distinct CpG island methylator phenotype (CIMP). HLRCC tumors demonstrated an extensive and relatively uniform level of hypermethylation that showed some correlation with tumor size. SDHB-RCC demonstrated a lesser and more varied pattern of hypermethylation that overlapped in part with the HLRCC hypermethylation. Combined methylation and mRNA expression analysis of the HLRCC tumors demonstrated hypermethylation and transcription downregulation of genes associated with the HIF pathway, *HIF3A* and *CITED4*, the WNT pathway, *SFRP1*, and epithelial-to-mesenchymal transition and MYC expression, *OVOL1*. These observations were confirmed in the TCGA CIMP-RCC tumors. A selected panel of probes could identify the CIMP tumors and differentiate between HLRCC and SDHB-RCC tumors. This panel accurately detected all CIMP-RCC tumors within the TCGA RCC cohort, identifying them as HLRCC -like, and could potentially be used to create a liquid biopsy-based screening tool. The CIMP signature in these aggressive tumors could provide both a useful biomarker for diagnosis and a target for novel therapies.

## Introduction

Hereditary leiomyomatosis and renal cell carcinoma (HLRCC) is a familial cancer syndrome characterized by the development of cutaneous and uterine leiomyomas, and a highly aggressive form of type 2 papillary kidney cancer [1–4]. HLRCC is associated with germline mutation of the Krebs cycle enzyme gene, fumarate hydratase (*FH*), and the resulting tumors demonstrate loss of the remaining wild-type allele [5–7]. Loss of fumarate hydratase both impairs oxidative phosphorylation, promoting increased levels of aerobic glycolysis [8, 9], and increases levels of intracellular fumarate, that effect multiple pathways. Increased intracellular fumarate levels causes the succination and functional alteration of numerous proteins, including the inactivation of KEAP1 resulting in the constitutive up-regulation of the NRF2-antioxidant response element (ARE) pathway and inactivation the core factors responsible for replication and proofreading of mitochondrial DNA (mtDNA) resulting in both a significant decrease in mtDNA content and increased mtDNA mutation [10–12]. Furthermore, increased fumarate inhibits the activity of prolyl hydroxylases that degrade the HIFα transcription factor subunits, resulting in a pseudo-hypoxic state that up-regulates many of the genes necessary to maintain the higher levels of glycolysis required by these tumors [13, 14]. Germline mutation of the succinate dehydrogenase subunit genes, *SDHA*, *SDHB*, *SDHC* or *SDHD*, is associated with increased risk for paraganglioma (PGL), pheochromocytoma (Pheo), gastrointestinal stromal tumor (GIST) and renal cell carcinoma [15–18]. In a similar manner to HLRCC tumors, the loss of succinate dehydrogenase activity results in suppression of oxidative phosphorylation and increased intracellular succinate can inhibit the prolyl hydroxylases [14, 19]. Prolyl hydroxylases are members of the 2-oxoglutarate (2OG)-dependent dioxygenase enzyme family, all of which could be inhibited to differing degrees by increased intracellular fumarate or succinate.

The ten-eleven translocation methylcytosine dioxygenases (TETs) are 2OG-dependent dioxygenases involved in the maintenance of the epigenome and the process of removing newly occurring aberrant CpG methylation [20, 21]. A study using a HEK293T embryonic kidney fibroblasts treated with cell permeable versions of fumarate and succinate or with stable knockdowns of *FH* and *SDHA/B* demonstrated inhibition of both the prolyl hydroxylases and the TET enzymes [22]. Subsequent studies of PGLs/Pheos and GISTs with SDH complex subunit gene mutation demonstrated a CpG island methylator phenotype (CIMP) and identified a single PGL sample with the CIMP phenotype that demonstrated an *FH* mutation, which could not be confirmed as germline or somatic [23, 24] Furthermore, a comparative study demonstrated that the patterns of methylation associated with loss of succinate dehydrogenase activity were similar but not identical between different tumor tissue types, such as PGLs verses GISTs [24]. The cancer genome atlas (TCGA) analysis of renal papillary cell carcinoma identified 9 out of 161 sporadic papillary RCCs that demonstrated a CIMP phenotype and a significantly poor prognosis [25]. Five out of these 9 tumors demonstrated mutation of *FH*, including 4 tumors from patients with germline *FH* mutation gene who had not been previously identified as HLRCC syndrome patients. No tumors demonstrated either germline or somatic mutation of the SDH subunit genes. Recently, a study by Sun, et al. evaluated a cohort of 25 patients with FH-deficient RCC, of which 17 patients had a germline mutation of FH and 8 had somatic FH mutation [26]. Within this cohort, 20 tumors were evaluated for methylation using EPIC arrays that demonstrated the presence of CIMP in 80% of tumors. The methylation profile of these CIMP positive FH-deficient RCC clustered with the TCGA CIMP-RCC tumors in a combined analysis with all TCGA renal tumors [26].

Although an increased risk of RCC is associated with germline mutations of the FH or the SDH subunit genes, the relative penetrance of this feature can be low. This means that patients can present with no evidence of family history or obvious additional syndromic features and be mistaken for sporadic cases as was the case for 4 tumors in the TCGA analysis [25]. For both HLRCC and SDH-RCC tumors a distinct histologic presentation can be observed and for HLRCC tumors several immunohistochemical markers are available [2, 27–30]. Yet, the addition of further biomarkers, such as specific hypermethylation signatures, that rely on standard, reproducible technologies would be very beneficial.

This study was designed to evaluate and compare the genome-wide levels of methylation within kidney tumors resulting from familial syndromes including HLRCC, SDH complex mutation (referred to herein as SDHB-RCC), von Hippel–Lindau syndrome (VHL) associated with germline mutation of *VHL*, hereditary papillary renal cell carcinoma (HPRC) associated with germline mutation of *MET*, and Birt-Hogg-Dubé syndrome (BHD) associated with germline mutation of *FLCN*. Furthermore, to evaluate the effect of the observed hypermethylation on gene expression within the HLRCC tumors.

## Materials and methods

### Sample acquisition

Archival formalin-fixed paraffin-embedded (FFPE) tumor tissues were selected from patients seen at the Urologic Oncology Branch (UOB) of the National Cancer Institute (NCI), National Institutes of Health (NIH) for clinical assessment and surgical resection. Tissue procurement and use was approved by the Institutional Review Board of the National Cancer Institute on either the NCI-97-C-0147 or NCI-89-C-0086 protocols and all patients provided written informed consent. Germline mutation status of patients was evaluated using targeted gene mutation assays performed by a CLIA-certified diagnostic laboratory. Samples were reviewed by a pathologist and regions of either characteristic tumor or normal histomorphology were enriched from paraffin blocks by needle punch. These tissue cores were lysed and processed to extract genomic DNA (gDNA) as previously described [31].

### Infinium 450 K Methylation assay for FFPE samples

Bisulfite conversion was performed on all samples using 250 ng of gDNA using the EZ DNA Methylation kit (Cat# 5004, Zymo Research) according to manufacturer’s recommendations. In brief, gDNA was denatured by addition of M-Dilution buffer, containing NaOH, and incubated at 37°C for 15 minutes. Sodium bisulfite-based CT-conversion reagent was freshly prepared and added to the denatured DNA samples before they were incubated for 16 hours at 50°C in a thermocycler with 30 second 95°C denaturation steps every hour. Following bisulfite conversion, DNA was bound to a Zymo-Spin™ IC Column, washed with M-Wash Buffer, desulphonated using M-desulphonation reagent for 20 minutes at room temperature and washed again with M-Wash Buffer. Bisulfite-converted DNA was eluted using 10 μL of M-Elution Buffer. Bisulfite-converted DNA originating from the paraffin embedded blocks was additionally restored using Infinium HD FFPE DNA Restore Kit (Cat#WG-321-1002, Illumina) following the manufacturer’s recommendations. This process is designed to restore potentially degraded FFPE DNA to improve amplification by the Infinium whole-genome amplification protocol. After DNA restoration, the Infinium HumanMethylation450 BeadChip assay was performed as previously described [32]. In brief, ~8 μl of bisulfite-converted DNA was initially processed in a whole-genome amplification reaction and subsequently enzymatically fragmented, precipitated, and re-suspended in hybridization buffer. All subsequent steps were performed using the standard Infinium protocol (User Guide part #15019519 A). Fragmented DNA was dispensed onto the HumanMethylation450 BeadChips and hybridization was performed in a hybridization oven for 20 hours. After hybridization, the arrays were processed through a primer extension and an IHC staining protocol to allow detection of a single-base extension reaction. Finally, BeadChips were coated and then imaged on an Illumina iScan. Methylation β values were calculated using BeadStudio software and any value associated with a p-value greater than or equal to 0.01 was removed and coded as NA for Not Applicable (Illumina, Inc.). All data was uploaded to the GEO repository with the GEO accession number GSE126441 (https://www.ncbi.nlm.nih.gov/geo/). For further analysis, any probe with an NA value within the core cohort of cohort of 33 tumor samples and 10 associated normal samples was removed resulting in probes being assessed.

### Cluster analysis

Unsupervised hierarchical cluster analysis using Euclidean distance was calculated using the Gene Cluster 3.0 software (http://bonsai.hgc.jp/~mdehoon/software/cluster/software.htm) and visualized using Java TreeView software (http://jtreeview.sourceforge.net/).

### *In-silico* Ingenuity-based pathway analysis

Pathway Analysis was performed using the Ingenuity Systems Interactive pathway analysis of complex ‘omics data software (IPA - http://www.ingenuity.com/) using the core analysis workflow. This provided data on the statistical enrichment of genes associated with disease, molecular and cellular function.

### RNAseq analysis of HLRCC tumors

Selected RNAseq data was acquired from a previous published analysis, Crooks *et al*. 2021, and all methodologies are described within that publication [12].

### TCGA analysis

All TCGA data was acquired from the National Cancer Institute’s Genomic Data Commons (https://gdc.cancer.gov/) using the data portal and, in part, the legacy archive. Annotation for the samples was acquired from the previously published study by Ricketts *et al*. 2018 [33].

## Results

### Methylation patterns in kidney tumors associated with genetically defined inherited kidney cancer syndromes

A cohort of 34 tumor samples and 11 associated normal samples consisting of 15 HLRCC syndrome renal tumors from the 12 individual patients with 4 associated normal samples, 6 *SDHB* mutation-related renal tumors with 4 associated normal samples, 5 VHL syndrome renal tumors with 3 associated normal samples, 4 HPRC syndrome renal tumors and 4 BHD syndrome renal tumors was selected for evaluation with Illumina HumanMethylation450 BeadChip arrays (GSE126441). These 31 patients consisted of 17 males and 14 females with surgeries occurring at between 19 and 63 years old with an average age at surgery of 40.5 years old. The early onset of disease in these patients is consistent with inherited RCC susceptibility syndromes. All patients had confirmed germline mutations in their respective syndromic genes including missense, nonsense, splice site, and frameshift point mutations as well as both partial and complete gene deletions (S1 Table).

**Fig 1:**
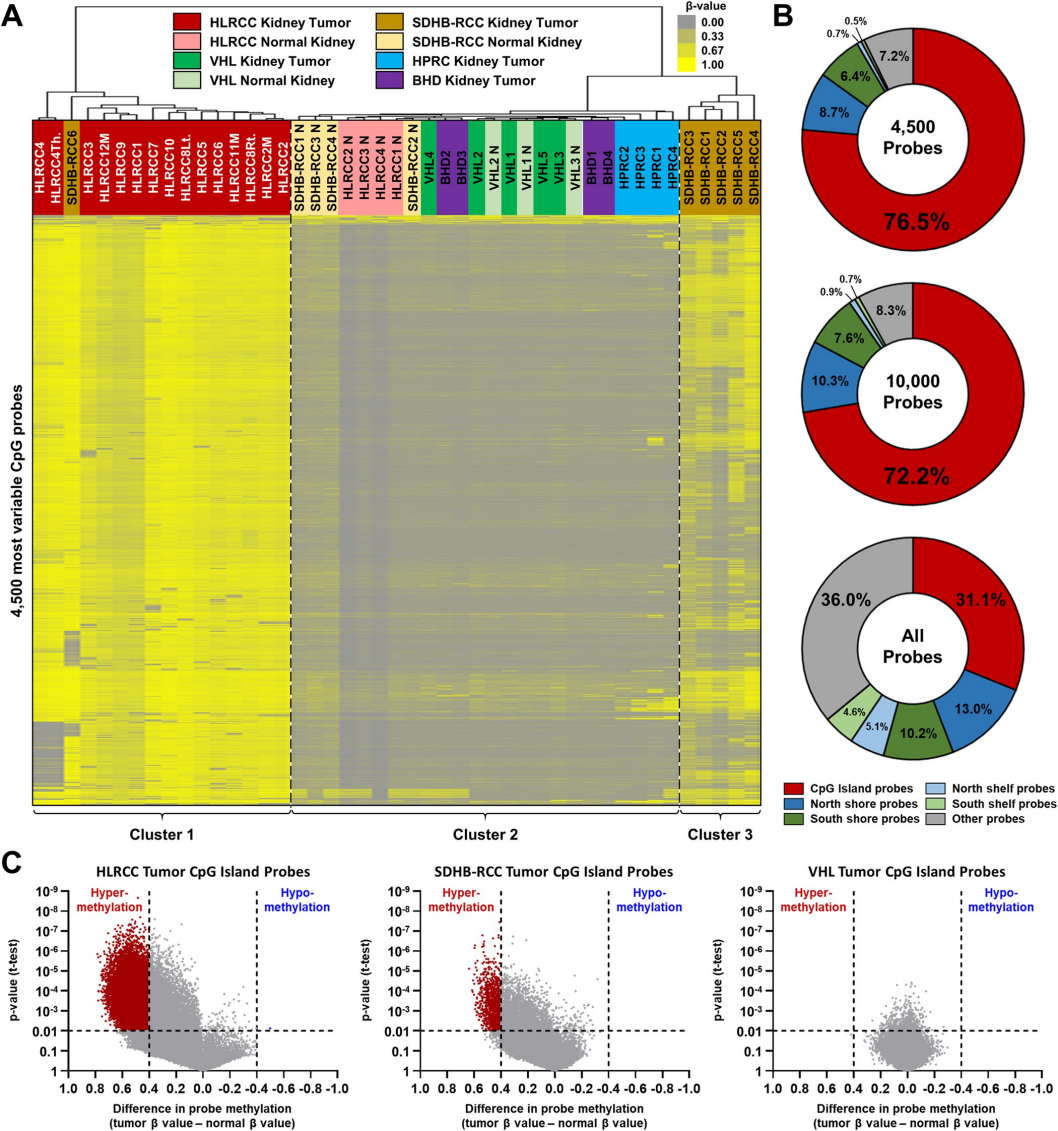
Methylation patterns in kidney tumors associated with inherited syndromes. A) Unsupervised hierarchical clustering using Euclidean distance and average linkage was performed using the β-values for the most variable 4,500 Illumina HumanMethylation450 BeadChip array probes (representing approximately 1% of all probes) across all the renal tumors derived from patients with germline mutations associated with kidney cancer susceptibly syndromes and their associated normal DNAs. This produced three distinct clusters including cluster 1 that contained all the HLRCC tumors and a single SDHB-RCC tumor (cluster 1) and cluster 3 containing the rest of the SDHB-RCC tumors. B) The Illumina HumanMethylation450 BeadChip array probes are spread throughout the genome and their positions relative to CpG islands were represented in the All Probes pie-chart. The 4,500 and 10,000 most variable array probes were significantly enriched for probes within CpG islands probes. C) Volcano plots were used to evaluate CpG island probe methylation status for the 4 tumor/normal samples pairs for HLRCC or SDHB-RCC patients and the 3 tumor/normal samples pairs for VHL patients. Probes with an average tumor difference in β-value of either 0.4 or -0.4 in comparison to the normal with a p-value of less than 0,01 were considered hyper- or hypomethylated respectively.

Illumina HumanMethylation450 BeadChip array analysis was performed on all samples and β-values were calculated for each probe. The 4,500 most variable probes across all 45 samples were selected to provide a profile for each sample, that represented approximately 1% of all probes, and assessed by unsupervised hierarchical clustering using Euclidean distance and average linkage (Fig 1A, S2 Table). In cluster 1 the probes had high β-values and consisted of all the HLRCC tumors and a single *SDHB* tumor, SDHB-RCC6, that was not distinctly different to the surrounding HLRCC tumors (Fig 1A). Both HLRCC2 and HLRCC4 primary tumors clustered next to their respective metastasis (HLRCC2M) and thrombus (HLRCC4Th.), suggesting a consistent, maintained methylation pattern. The metastatic samples did not cluster together and the two independently arising tumors from the same patient (HLRCC8) also showed differences. Conversely, cluster 2 probes had low β-values and contained all the associated normal samples and all the tumors from the VHL, BHD and HPRC patients (Fig 1A). Notably the VHL normal samples clustered more closely with their associated tumor than with the other VHL normal, suggesting that any methylation differences were patient specific rather than tumor specific. The majority of the HLRCC normals clustered together, as did the SDHB-RCC normals, suggesting that some mild methylation signature light be shared in the normal tissues. A full examination of this would require comparison to normal kidney tissue from genetically unaffected individuals and was not within the scope or aims of this project. Cluster 3 contained the majority of the SDHB-RCC tumors and had a mixture of probes with high and low β-values, suggesting a methylated phenotype that is less extensive and more variable than in the HLRCC tumors (Fig 1A). The 4,500 most variable probes demonstrated significant enrichment for CpG island probes, and this was maintained when the analysis was expanded to 10,000 probes (Fig 1B).

The true extent of the hypermethylation was evaluated using volcano plots to evaluate the differences between normal and tumor in the paired samples. The 4 paired HLRCC samples had a huge abundance of hypermethylated probes in all regions, but also showed evidence for hypomethylation as well (Fig 1C, S1A Fig in S1 File). The largest number of significantly hypermethylated probes were in the CpG islands (13,367/148,128, 9.02%), followed by the CpG shores and shelves (6,217/153,784, 4.04%), with the least in the non-CpG (Other) regions (2,985/167,862, 1.78%). Conversely, only a single significantly hypomethylated probe was seen in the CpG island with greater numbers in the CpG shores and shelves (77) and the non-CpG (Other) regions (435). The 4 paired SDHB-RCC samples demonstrated a clear pattern of hypermethylation in all regions but less probes reached statistical significance, consistent with the more variable natural of the hypermethylation (Fig 1C, S1B Fig in S1 File). The largest number of significantly hypermethylated probes were in CpG islands (1,008) and the least in the non-CpG (Other) regions (111); no significantly hypomethylated probes were seen. In comparison, the 3 paired VHL samples demonstrated no hyper- or hypomethylation and no significantly altered probes were seen in the any region (Fig 1C, S1C Fig in S1 File).

### Analysis of the functional effect of hypermethylation in HLRCC kidney tumors on the transcriptome

Tumor-specific hypermethylated (TS-Hyper) probes were identified for the HLRCC tumors by selecting probes with β-values of ≤0.2 in all 4 HLRCC associated kidney normal tissues and an average HLRCC tumor (n = 15) β-value ≥0.4 higher than the average HLRCC associated kidney normal tissue (n = 4) β-value. This identified 10.2% of the CpG island probes (15,089/148,128), 3.24% of the CpG shores and shelves probes (4,982/153,784), and 0.97% of the probes in non-CpG (Other) regions (1,634/167,862) (S3 Table). Approximately three-quarters (74.9%) of the TS-Hyper CpG island probes were associated with one or more genes and potentially methylated genes were defined as having 2 or more TS-Hyper CpG island probes. This highlighted 2,439 potentially methylated genes and RNAseq data was available to evaluate 2151 of these genes (S3 Table). The RNAseq data compared 5 primary and 8 metastatic tumors from 7 of these patients with 9 kidney normal tissues, 5 from HLRCC patients (including HLRCC2, HLRCC3, and HLRCC4) and 4 from unaffected patients. This identified 352 downregulated methylated genes that demonstrated both 2 or more TS-Hyper CpG island probes and a tumor:normal expression ratio of <0.6667 with a p-value <0.05, indicating a degree of concordant downregulation in gene expression (S3 Table).

**Fig 2:**
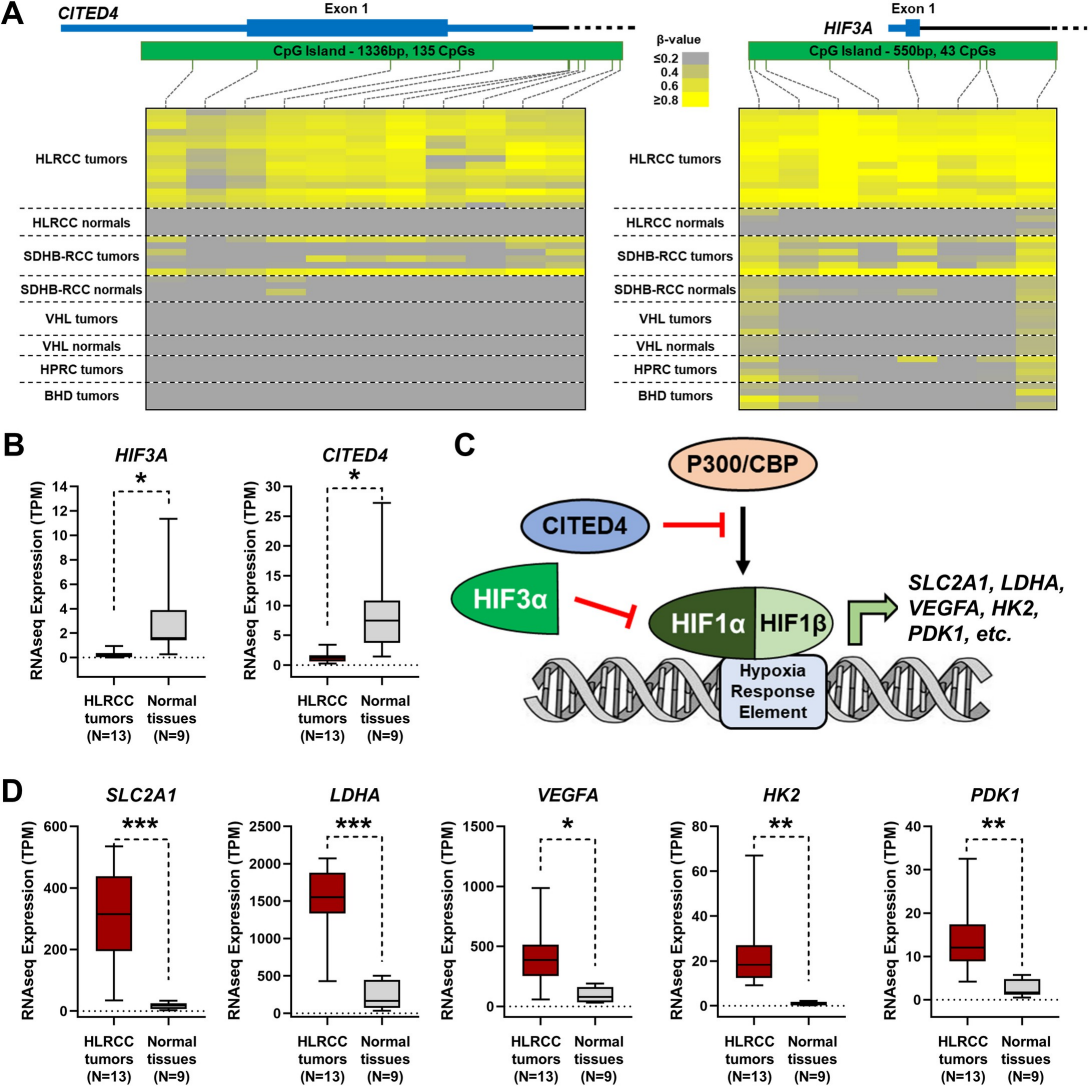
Hypermethylation of HIF pathway genes in HLRCC tumors. A) Methylation β-value heatmaps for all the Illumina HumanMethylation450 BeadChip array probes within the CpG islands of the *CITED4* and *HIF3A* genes. B) mRNA expression graphs for *CITED4* and *HIF3A* comparing 13 HLRCC tumors with 9 normal tissues (5 from HLRCC patients and 4 from unaffected patients). C) A schematic representation of inhibition of the HIF pathway by either HIF3A competitively binding the hypoxia response elements or CITED4 inhibiting the HIF1 co-activators, P300/CBP. Removal of both genes increases the potential activity of HIF1 and the expression of downstream targets, such as *SLC2A1* (GLUT1) and *LDHA*. D) mRNA expression graphs for selected downstream targets of the HIF pathway.

**Fig 3:**
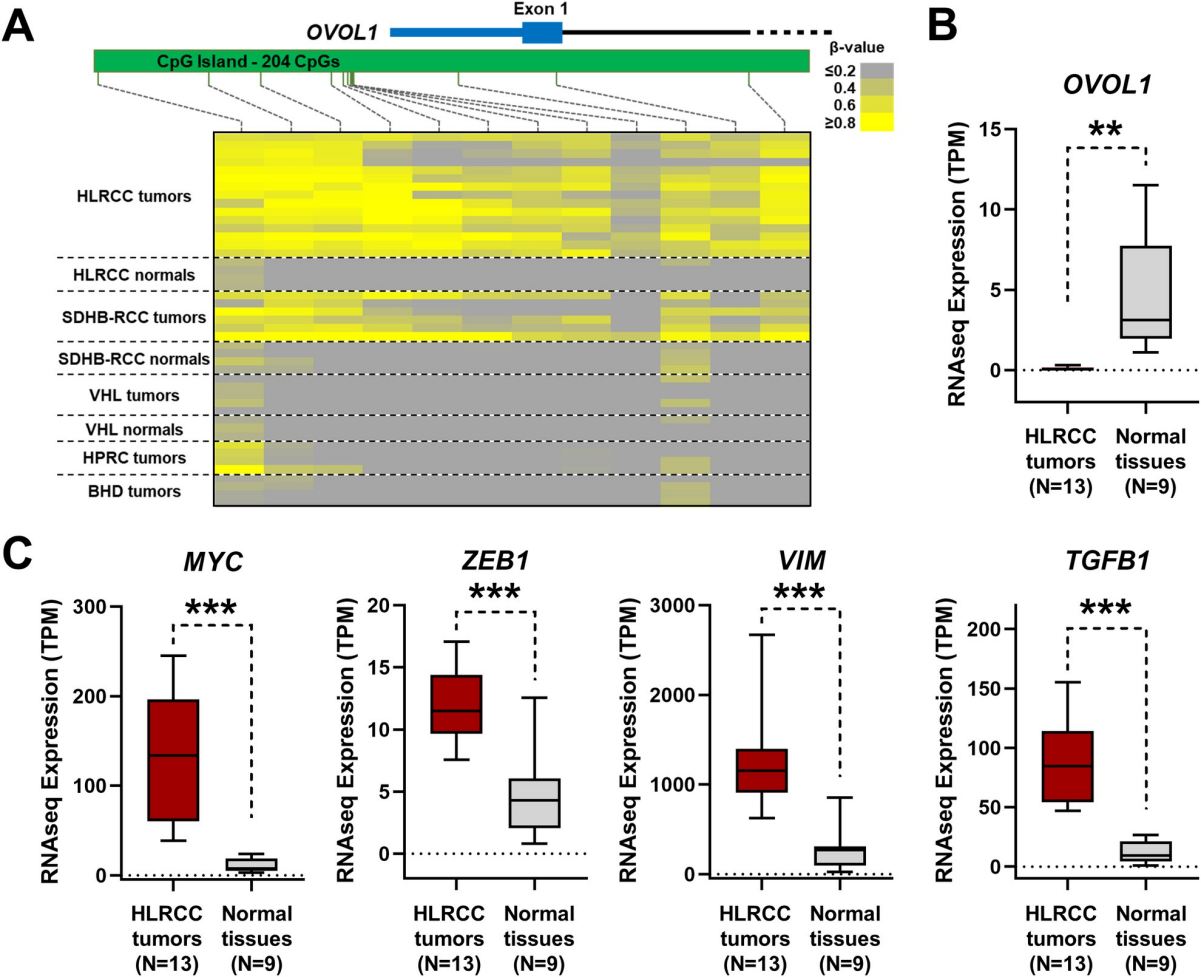
Hypermethylation of *OVOL1* in HLRCC tumors. A) Methylation β-value heatmaps for all the Illumina HumanMethylation450 BeadChip array probes within the CpG islands of the *OVOL1* gene. B) mRNA expression graphs for *OVOL1* comparing 13 HLRCC tumors with 9 normal tissues (5 from HLRCC patients and 4 from unaffected patients). D) mRNA expression graphs for *OVOL1* downstream genes including *MYC* and EMT genes, such as *ZEB1* and *VIM*.

*In-silico* Ingenuity-based pathway analysis of these 352 genes demonstrated an enrichment for genes association with cancer as a disease, cell death and survival as a biological function, and β-catenin-related regulation (S2 Fig in S1 File). The *SFRP1* and *DKK1* genes that are elements of the WNT pathway that regulates β-catenin have been shown to be methylated in the TCGA analysis of CIMP tumors that include tumors with germline or somatic *FH* mutations. The HLRCC tumors showed methylation-associated downregulation of *SFRP1*, *FRZB* (previously known as *SFRP3*), and *WNT3* mRNA expression, all components of the WNT pathway, but no downregulation of *DKK1* (S3 Fig in S1 File). Dysregulation of the hypoxia response pathway has been reported in HLRCC tumors. Two regulators of HIF1α showed methylation-associated mRNA downregulation, *CITED4* and *HIF3A*, and this correlated with the expected increased expression of HIF downstream targets, such as *SLC2A1*, *LDHA*, and *VEGFA* (Fig 2). HLRCC tumors have also been shown to demonstrate increased *MYC* expression and an increased EMT (epithelial-to-mesenchymal transition) signature. *OVOL1* and *OVOL2* encode transcription factors that suppress both MYC and EMT. The HLRCC tumors had significant methylation-associated mRNA downregulation of *OVOL1*, although *OVOL2* had no methylation or downregulation, and this correlated with the expected increased expression of *MYC* and the markers of EMT including *ZEB1*, a transcriptional target of OVOL1 (Fig 3). Notably, *CDKN2A* promoter hypermethylation has also been associated with the TCGA CIMP tumors and this was true for this cohort of HLRCC tumors but showed no correlation with reduced mRNA expression (S4 Fig in S1 File). Thus, *CDKN2A* methylation could be a marker of HLRCC tumors but does not seem to have a functional effect. In fact, the expression of *CDKN2A* was increased in the tumors and this was true of several other genes that also demonstrated TS-Hyper CpG island probes (S3 Table). It is likely that these genes are being transcriptionally activated by several other processes and that the hypermethylation is so extensive within these tumors that it can be present to a degree within the CpG islands of genes that remain transcriptionally active.

### Hypermethylation in SDHB-RCC kidney tumors and comparison to other SDH-Deficiency tumors

**Fig 4:**
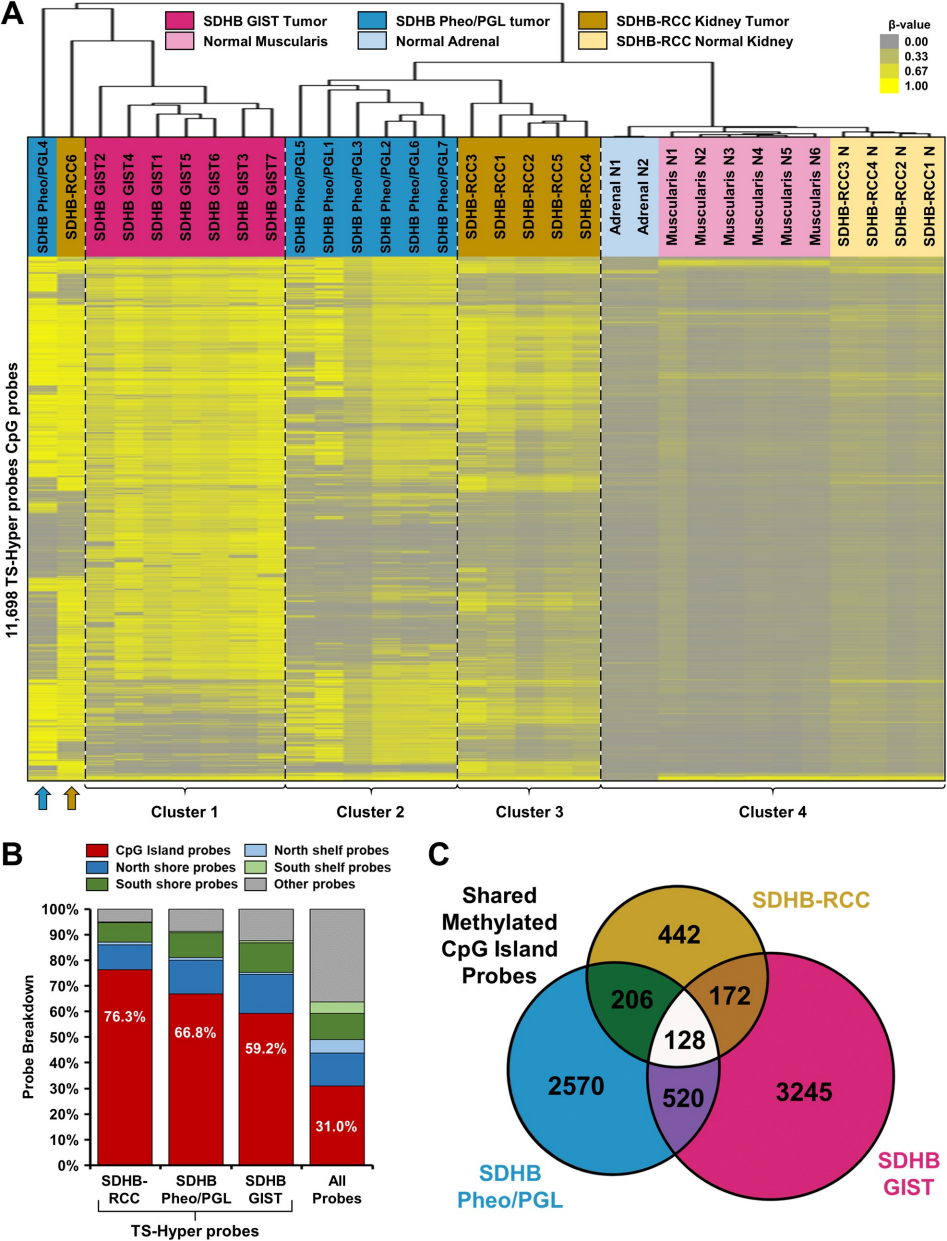
Comparative methylation patterns in different SDHB mutant tumor types. A) Unsupervised hierarchical clustering using Euclidean distance and average linkage was performed using the β-values for the 11,698 Illumina HumanMethylation450 BeadChip array probes identified as being TS-Hyper probes in either SDHB-RCC, SDHB Pheo/PGL, or SDHB GIST. Three distinct tumor-type specific clusters were created with two outliers, consisting of 1 SDHB-RCC (marked with a gold arrow) and 1 SDHB-Pheo/PGL (blue arrow). B) The relative frequencies of the locations of the TS-Hyper probes identified in the SDHB-RCC, SDHB Pheo/PGL, and SDHB GIST tumors in comparison to all the Illumina HumanMethylation450 BeadChip array probes. All tumor types show enrichment for probes within CpG islands probes. C) A Venn diagram showing the overlap of CpG island-based TS-Hyper probes identified in the SDHB-RCC, SDHB Pheo/PGL, and SDHB GIST tumors.

Hypermethylation has been previously reported in other manifestations of germline SDHB mutation, including paraganglioma (PGL), pheochromocytoma (Pheo) and the gastro-intestinal stromal tumors (GIST) associated with SDH subunit gene mutation [23, 24]. Hypermethylation of the SDHB-RCC demonstrated some overlap with the HLRCC tumors and publicly available data was obtainable for Illumina HumanMethylation450 BeadChip analysis of 7 SDHB-mutant Pheo/PGL tumors with 2 normal tissue samples (GSE43298) and 7 SDHB-mutant GISTs with 6 normal tissue (muscularis) samples (GSE34387). TS-Hyper probes were identified for the SDHB-RCC tumors in this cohort and the two external cohorts (Pheo/PGL and GIST) by selecting probes with β-values of ≤0.2 in all normal tissue associated with each cohort and an average tumor β-value ≥0.4 higher than the average normal tissue β-value. This identified 11,698 probes in total, 1,248 in SDHB-RCC, 5,128 in SDHB Pheo/PGL, and 6,881 in SDHB-GIST (15,089/148,128, 10.2%), and clustering of these probes separated out the different tissue types (Fig 4A, S4 Table). Cluster 1 contained all the SDHB-GIST samples that showed a uniform pattern of high methylation in a larger number of probes with considerable overlap with the methylated probes in both SDHB-RCC and SDHB Pheo/PGL. Cluster 2 contained the majority of SDHB Pheo/PGL tumors that demonstrated considerable variation between tumors and there was a more highly methylated outlier that clustered more closely to the SDHB GIST tumors. Cluster 3 contained the majority of SDHB-RCC tumors that maintained the variation between tumors previously observed with the highly methylated outlier SDHB-RCC6 that clustered more closely to the SDHB GIST tumors. Finally, cluster 4 contained all the normal tissues and some tissue specific methylation could be observed.

The outlier SDHB-RCC6 was diagnosed at 55, a little older than the average age for the 6 SDHB-RCC tumors of 39 years old but was not the oldest. The tumor was 1.8 cm in the greatest dimension, but 5 of the 6 SDHB-RCC tumors fell within a size range of 1.5–2.2 cm (S1 Table). While the outlier SDHB Pheo/PGL4 was diagnosed at 28 with the average age for diagnosis of the SDHB Pheo/PGL tumors being 39 years old (range of 24–59 years old) [23]. No particularly unusual clinical feature was associated with this sample. Neither outlier demonstrated any evidence for why they demonstrated such a different level of hypermethylation. Additional somatic mutations within these tumors could further enhance the hypermethylation and investigation of a reasonable number of these outliers could highlight potential modifiers of hypermethylation.

All three tumor types demonstrated enrichment of hypermethylation in the CpG island probes consistent with CIMP and SDHB-RCC showed the greatest enrichment (76.3%) (Fig 4B). Although the methylation patterns demonstrated a reasonable amount of overlap between the tumor types (Fig 4A), only a small number of CpG island probes that passed the strict TS-Hyper probe criteria were shared between all tumor types (n = 128) (Fig 4C, S4 Table). A further 378 TS-Hyper probes within CpG islands were shared between SDHB-RCC and one other tumor type (Fig 4C). The combined 506 probes represented 256 genes with 92 genes having at least two probes (S4 Table). The effect of hypermethylation on the expression of these genes was not available for the SDHB-RCC tumors.

### Identification of CIMP marker probes for the hypermethylation profiles in the HLRCC and SDHB-RCC tumors and comparisons to the TCGA CIMP tumors

**Fig 5:**
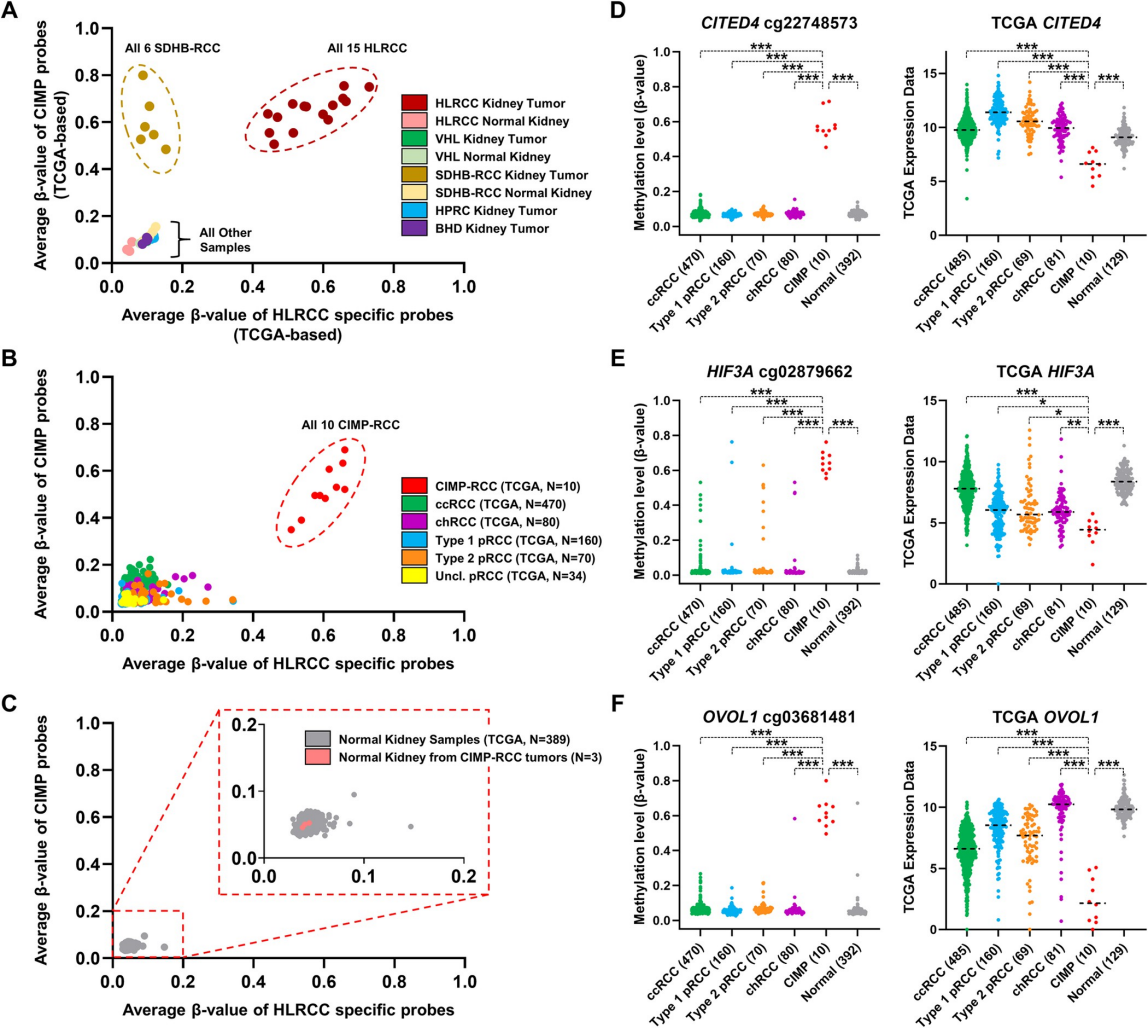
Confirmatory analysis of HLRCC hypermethylation patterns in TCGA RCC tumors. A set of 16 CIMP probes and 15 HLRCC specific probes that were highly methylated for these two criteria were identified from this cohort that were also present in the TCGA analysis of RCC. A) The average β-values for both probe sets were calculated for each tumor in this cohort and graphed to show a clear differentiation between CIMP tumors and the other tumors and between HLRCC and SDHB-RCC tumors. B) Analysis of 824 RCCs from the TCGA RCC cohort clearly distinguished the previously identified CIMP-RCC tumors within this cohort. C) Analysis of 392 associated normal kidney samples from the TCGA RCC cohort showed no methylation in these probes sets, including in 3 samples from patients with CIMP-RCC tumors. D-F) A representative Illumina HumanMethylation450 BeadChip array CpG island probe for each of the *CITED4*, *HIF3A* or *OVOL1* genes was evaluated for methylation and comparisons were made between the CIMP-RCC tumors and all other tumor types and normal. Similarly, the expression of *CITED4*, *HIF3A* or *OVOL1* was compared between the CIMP-RCC tumors and the other tumor types and normal. All statistics were t-tests and p-values were defined as * <0.05, ** <0.001, *** <0.0001.

CIMP-specific probes were selected that had β-values >0.5 in all 21 HLRCC/SDHB-RCC tumors and <0.2 in all 24 non-CIMP tumor and normal samples. This identified 54 CpG island probes and 25 probes were selected by their p-value calculated by comparing CIMP to non-CIMP samples (S5 Table). HLRCC-specific probes were selected that had β-values >0.5 in at least 13 out of the 15 HLRCC tumors and <0.2 in all other tumor and normal samples, including SDHB-RCC tumors, and this identified exactly 25 CpG island probes (S5 Table). The averages of the 25 CIMP-specific and 25 HLRCC-specific probes were calculated and plotted in a scatter graph for all samples within this cohort (S5 Fig in S1 File). All 15 HLRCC tumors clustered in the top right of the graph and all 6 SDHB-RCC tumors clustered in the top left of the graph (S5 Fig in S1 File). All other kidney tumor and all associated normal samples clustered in the bottom left of the graph demonstrating very little methylation (S5 Fig in S1 File). This 50-probe panel could be used diagnostically to evaluate tumors and to evaluate existing datasets such as the TCGA. The TCGA analysis of RCC had already identified a small subset of CIMP tumors and these tumors were associated with germline and somatic *FH* mutation and low expression of the *FH* mRNA. Additionally, a previous study of hypermethylation profile in FH-deficient RCC tumors, associated with both germline and somatic *FH* mutations, has shown a very strong correlation with these samples [26]. Unfortunately, the TCGA has used both the Infinium® HumanMethylation27 and HumanMethylation450 BeadChips in different samples and only analyzed probes that overlapped between the two chips. To account for this, CIMP-specific and HLRCC-specific probes were reselected based on the probes available in the TCGA data. This identified 16 CIMP-specific probes that had β-values >0.5 in at least 18 out of the 21 HLRCC/SDHB-RCC tumors and 15 HLRCC-specific probes that had β-values >0.5 in at least 10 out of the 15 HLRCC tumors (S5 Table). For both probes sets the remaining samples had β-values <0.2. Plotting the averages of these TCGA-restricted 31-probe set demonstrated the same pattern as the 50-probe set with a slightly wider dispersal of CIMP tumors but equally tight clustering of the non-CIMP samples (Fig 5A). Analysis of the 824 TCGA RCC available, that included all the major subtypes of RCC, clearly identified the previously defined 10 TCGA CIMP-RCC samples and they showed a signature consistent with the HLRCC tumors (Fig 5B). All other tumors demonstrated minimal levels of methylation. Furthermore, analysis of the 392 normal tissues available in the TCGA data, including normal tissues from 3 TCGA CIMP-RCCs, all demonstrated no methylation in this probe set (Fig 5C). This confirmed the specificity of this type of methylation pattern analysis in a large cohort of sporadic tumors.

As the CIMP-RCC tumors in the TCGA were identified as having HLRCC like methylation patterns, they were used as a confirmatory cohort for the methylation-associated gene expression downregulation seen in this cohort’s HLRCC tumors. Selected CpG island probes for *SFRP1*, *CITED4*, *HIF3A*, *OVOL1*, and *CDKN2A* all showed significantly higher methylation in the TCGA CIMP-RCCs in comparison to the other tumors and normal tissues (Fig 5D–5F, S6 Fig in S1 File). Methylation in these genes was matched with expression downregulation in the available data for 805 TCGA CIMP-RCCs in comparison to 129 normal tissues for *SFRP1*, *CITED4*, *HIF3A*, and *OVOL1* (Fig 5D–5F, S6 Fig in S1 File). Notably, *CITED4* mRNA was only downregulated in the TCGA CIMP-RCC (Fig 5D). *SFRP1* mRNA was downregulated in all tumor subtypes in a similar manner to TCGA CIMP-RCC (S6 Fig in S1 File). *HIF3A* mRNA was downregulated in papillary (p)RCC and chromophobe (ch)RCC with TCGA CIMP-RCC showing a greater downregulation (Fig 5E). *OVOL1* mRNA was mildly downregulated in ccRCC with TCGA CIMP-RCC showing a greater downregulation and a corresponding increase in *MYC* expression (Fig 5F, S6 Fig in S1 File). *CDKN2A* mRNA was upregulated in TCGA CIMP-RCC in comparison to normal tissues, as it was for all the RCC tumor subtypes (S6 Fig in S1 File). This data confirmed the methylation driven downregulation of genes controlling the HIF pathway and EMT was present in the TCGA CIMP-RCC samples in a similar manner to the HLRCC tumors in this cohort.

### Comparative hypermethylation levels in HLRCC and SDHB-RCC tumors

**Fig 6:**
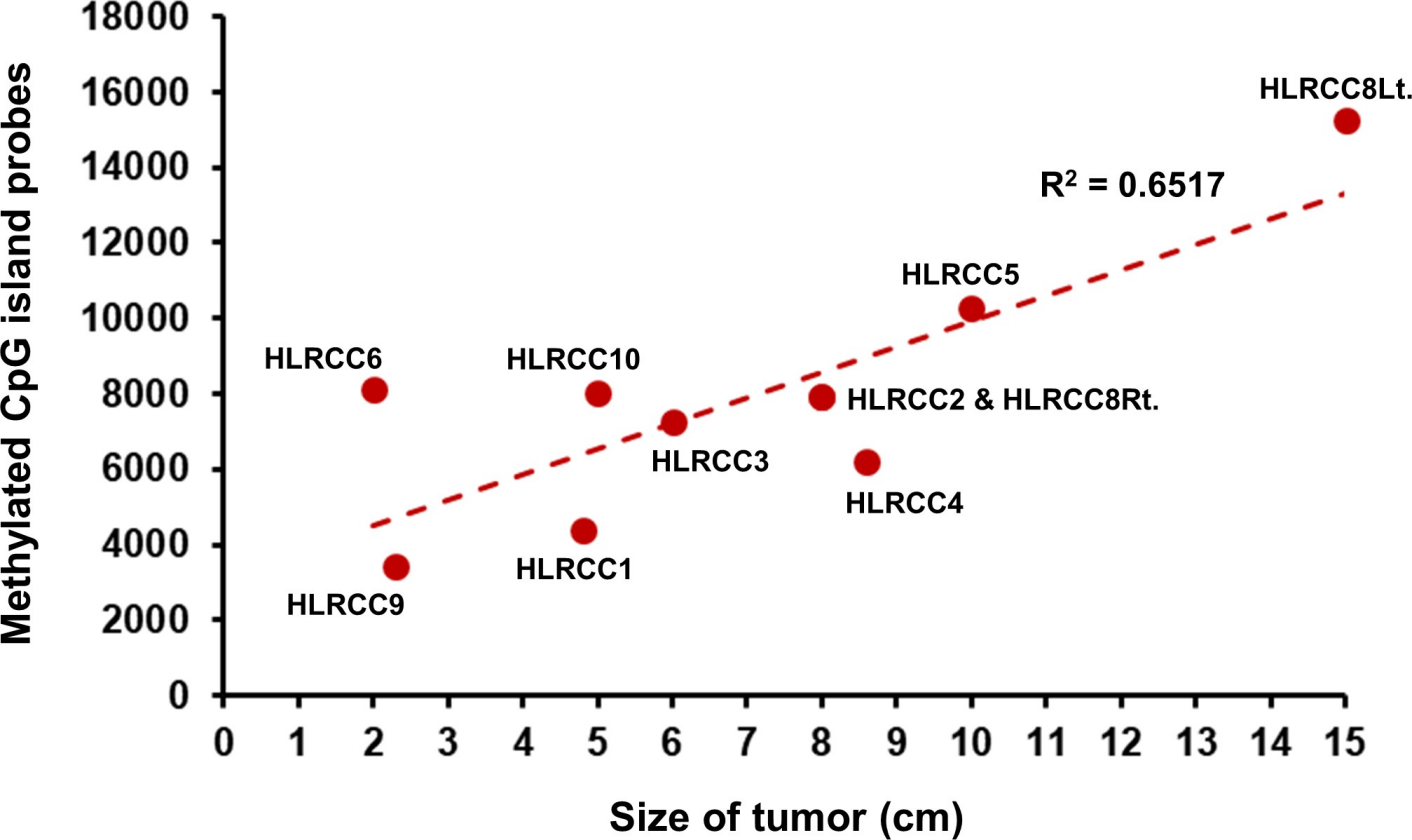
Relative hypermethylation compared to size in HLRCC tumors. The general level of hypermethylation within each tumor was calculated by first identifying all CpG island probes with β-values less than 0.2 in all 11 associated normal samples (4x HLRCC normal, 4x SDHB-RCC normal, and 3x VHL normal) were identified (n = 31287). Then, for each HLRCC primary kidney tumor the number of these probes where the β-value was greater than 0.5 was calculated and used as a representative level of hypermethylation. This was plotted against tumor size and a linear trendline was added.

To evaluate the general level of hypermethylation within each tumor all CpG island probes with β-values less than 0.2 in all 11 associated normal samples (4x HLRCC normal, 4x SDHB-RCC normal, and 3x VHL normal) were identified (n = 31287) (S6 Table). For each HLRCC or SDHB-RCC primary kidney tumor the number of these probes where the β-value was greater than 0.5 was calculated and used as a representative level of hypermethylation (S6 Table). The HLRCC primary tumors ranged in value from 3,448 to 15,258 (11.0% to 48.8% of the selected probes) and a linear correlation with tumor size (n = 10) was observed, while no correlation with patient age at diagnosis (n = 11) was seen (Fig 6 and S7 Fig in S1 File). SDHB-RCC tumors ranged in value from 520 to 9,642 (1.7% to 30.8%) and no correlation with tumor size (n = 6) was observed, but there was a weak exponential correlation with age at diagnosis (n = 6). For both HLRCC and SDHB-RCC the number of primary tumors was relatively small, limiting the extent of this analysis, but there is an obvious difference between the tumor types (S7 Fig in S1 File).

## Conclusions

Methylation analysis of both HLRCC and SDHB-RCC tumors identified a CpG island methylator phenotype (CIMP) in both tumor types that demonstrated a degree overlap in the probes that were hypermethylated. HLRCC tumors showed a greater number hypermethylated probes in comparison to the SDHB-RCC tumors and showed less variation in the pattern of hypermethylation within the probes selected in this study. A major caveat in dealing with rare tumors such as HLRCC and SDHB-RCC tumors is that only a small number of tumors are available for analysis in comparison to sporadic disease. While this study does show distinct contrast in hypermethylation between the CIMP-RCC tumors and the other RCCs, it is also supported by previously published work. In the case of the HLRCC tumors, this CIMP phenotype has been seen within a previous study of 20 FH-deficient tumors, both with germline and sporadic *FH* mutations, and within the 10 CIMP-RCCs tumors identified within the Cancer Genome Atlas (TCGA) analysis of RCC [26, 33]. Similarly, paragangliomas, pheochromocytomas and GISTs associated with germline mutation of *SDHB*, *SDHC* and *SDHD* have been previously shown to exhibit CIMP in comparison to tumors caused by mutations in other genes [23, 24]. This study confirms that CIMP is also present in the *SDHB* mutant RCC, although no samples were available for *SDHC* or *SDHD* mutant RCCs. Comparison of the data in these previous studies with the cohort herein demonstrated a stronger hypermethylation signature was seen in the GIST and pheochromocytoma/paraganglioma samples in comparison to the SDHB-RCC samples and that the pheochromocytoma/paraganglioma samples showed a variable degree of hypermethylation, like the SDHB-RCCs. A recent case study also reported the presence of the known somatic mutation of *IDH2* (p.R172M) in multiple RCCs that demonstrated increased levels of 2-hydoxygluatarate and the resultant CpG island methylator phenotype [34]. Thus, currently a CIMP phenotype can be observed in RCC due to the alteration of three separate Krebs cycle enzymes, fumarate hydratase, succinate dehydrogenase, and isocitrate dehydrogenase.

The mechanism of this hypermethylation has previously been elucidated as it has been shown that the increased levels of fumarate or succinate oncometabolites inhibit the action of the ten-eleven translocation methylcytosine dioxygenase (TET) enzymes responsible for the maintenance of the epigenome and removal of aberrant CpG methylation [20–22]. A study using a HEK293T embryonic kidney fibroblasts treated with cell permeable versions of fumarate and succinate demonstrated similar inhibition of the TET enzymes for both metabolites [22]. The overall greater degree of hypermethylation in HLRCC tumors compared to the SDHB-RCC tumors could be related to several factors. It has been shown that germline *FH* mutation may raise succinate levels as well as fumarate levels possibly creating a greater effect on the TET enzymes [14]. Also, HLRCC tumors may either create more fumarate or retain a higher concentration of fumarate within the tumor cells that the SDHB-RCC tumors can for succinate. Alternatively, the increased levels of hypermethylation within HLRCC tumors could be either more beneficial, necessary, or tolerable in conjunction with the other changes induced by *FH* mutation than with *SDHB* mutation, allowing for a greater enrichment of hypermethylation. The degree of hypermethylation in HLRCC tumors demonstrated a constant increase as the tumor size increased, that was not observed in the SDHB-RCC tumors. This may reflect a more constant and effective inhibition of the TET enzymes in HLRCC compared to SDHB-RCC tumors. Notably, the degree of CIMP in the SDHB GIST and SDHB Pheo/PGL tumors was greater than in SDHB-RCC demonstrating that increased succinate levels can have greater effects on hypermethylation [23, 24]. It can be assumed that the vast majority of the hypermethylation that is occurring is not directly influencing tumorigenesis, similar to the large number of passenger mutations in a tumor with a hypermutator phenotype. Thus, a majority of the hypermethylation is occurring in places where it will neither benefit nor hinder tumor growth and this may be heavily dependent on the tissue the tumor is derived from. This is concordant with the overlap in the CIMP hypermethylation pattern between the kidney tissue derived HLRCC and SDHB-RCC tumors and the degree of variation observed between the different tumor types (RCC, GIST, and Pheo/PGL) associated with germline SDHB mutation. This is also reflected in the fact that a small subset of probes can be used to distinguish the CIMP tumors from the other RCC tumors both in this cohort and within the 800 RCC tumors in the TCGA cohort. This could prove an accurate and effective diagnostic test for CIMP tumors in patients and has the potential to differentiate between the causes of CIMP, as seen with the separation of HLRCC and SDHB-RCC tumors herein. It is possible to perform methylation analysis by liquid biopsy of either blood or urine to detect hypermethylation in cell free DNA [35–39]. A recent study combined cell-free methylated DNA immunoprecipitation and high-throughput sequencing (cfMeDIP–seq) from both patient plasma and urine to detect RCC in patients [38]. This included the detection of small (stage I/T1a), localized clear cell tumors [38]. This was achieved using a training set to identify differentially methylated regions, but the data from this study would already provide these regions for CIMP tumors. Early identification and evaluation of these tumors is crucial due to the rapid metastasis and poor outcomes that are associated with CIMP tumors, even very small ones [27, 33, 40]. Non-invasive techniques would be particularly useful for the surveillance of patients with germline alterations that require regular screening.

The HLRCC tumors demonstrated a very significant level of methylation in CpG island probes with ~10% of those probes showing increased methylation in comparison to the associated normal tissue. It is important to note that evidence for promoter methylation does not automatically translate to downregulation of the gene the CpG island is associated with. In some cases, the genes would not be expressed in that tissue and in other cases the hypermethylation would not be sufficient to alter gene expression. This highlights the importance of comparing the hypermethylation data with expression data. Several genes of interest were both hypermethylated and downregulated in the HLRCC tumors. SFRP1, a WNT pathway member, has been shown to be methylated and downregulated in the TCGA RCC cohort, including within the TCGA CIMP tumors, and has been associated with poorer outcome in ccRCC [33, 41–43]. This was confirmed within this study with several WNT pathway members, *SFRP1 FRZB*, and *WNT3*, being hypermethylated and downregulated. HIF1α stabilization is a known occurrence in HLRCC tumors and results in activation of the HIF response pathway [13]. *HIF3A* encodes a transcription factor capable of being a dominant-negative inhibitor of HIF1α and has been reported to be downregulated in ccRCC [44, 45]. *CITED4* encodes a transcriptional regulator that can bind to several proteins including CREB-binding protein (CBP)/p300 to inhibit its interaction with HIF1α and suppress the activation of the HIF pathway [46]. Both HIF3A and CITED4 were hypermethylated and downregulated in the HLRCC tumors consistent with enhancing the HIF1α-driven activation of the HIF pathway. *OVOL1* was hypermethylated and downregulated in the HLRCC tumors and encodes a transcription factor that has been shown to suppress expression of *MYC* and *ZEB1*, a transcriptional promoter of epithelial-to-mesenchymal transition (EMT) [47–50]. MYC has been shown to be upregulated in HLRCC tumors and drugs that downregulate MYC suppress the growth of HLRCC tumor cell lines [51]. HLRCC tumors have also been shown to demonstrate several markers of EMT including increased *ZEB1* expression and downstream markers of *ZEB1* upregulation, such as *VIM*, were upregulated in this cohort [52, 53]. Loss of *OVOL1* expression could significantly contribute to the aggressive nature of HLRCC tumors. All these observations were confirmed by analysis of the TCGA CIMP tumors in comparison to the remaining RCC tumor and normal samples within the TCGA cohort. *CDKN2A* hypermethylation is a common event in cancer and had been previously associated with the TCGA CIMP samples [33]. Notably, the HLRCC tumors confirmed the hypermethylation seen in the TCGA study but no effect on *CDKN2A* mRNA expression was observed with the hypermethylated tumors having a greater expression that the unmethylated normal samples. This provides a good example of why the mRNA data is so necessary.

Potentially, CIMP renal tumors could be therapeutically susceptible to de-methylation agents, such as 5-azacytidine or 5-aza-2′-deoxycytidine, that could reactivate these suppressed genes. A study treating patient-derived *IDH1* mutant glioma xenografts, that demonstrate a specific CIMP profile of their own, with the 5-azacytidine demethylating agent has already shown tumor regression [54]. The potential for this is under investigation as a Phase II trial of a DNA methyl transferase inhibitor, Guadecitabine (SGI-110), is currently underway to evaluate its effectiveness in hypermethylated tumors and is recruiting patients with HLRCC-associated kidney cancer (ClinicalTrials.gov Identifier: NCT03165721). Previous analysis has shown that the increased levels of fumarate or succinate within these tumors is preferentially derived from glutamine via incorporation into the Krebs cycle and that those metabolite levels could be potentially lowered by inhibition of the glutaminase enzyme, reversing some transient effects of increased metabolite levels but not the hypermethylation [9, 55]. Thus, combining 5-aza-2′-deoxycytidine treatment with a glutaminase inhibitor may provide a greater anti-tumorigenic than either treatment alone.

In conclusion, the CIMP observed within these HLRCC and SDHB-RCC tumors distinguished these tumors from other genetically defined familial kidney cancer syndrome associated tumors. Within HLRCC tumors, hypermethylation could be correlated with the downregulation of potential tumor suppressor genes. This specific CIMP profile could aid in the efficient diagnosis of these aggressive tumors by providing a non-invasive screening method and potentially be a therapeutic target.

## Supporting information

S1 Table Patient mutation and clinical data. (XLSX)

S2 Table 10,000 most variably methylated probes across all tumor samples. (XLSX)

S3 Table Tumor-specific hypermethylation probes identified in the HLRCC tumors. (XLSX)

S4 Table Tumor-specific hypermethylation probes identified in SDHB-RCC, SDHB Pheo/PGL, and SDHB GIST tumors. (XLSX)

S5 Table Selected methylated probe sets to identify and CIMP or HLRCC tumors. (XLSX)

S6 Table Relative tumor hypermethylation levels and association with size and age. (XLSX)

S1 File (DOCX)
