# Supplementary material for: Kidney tumors associated with germline mutations of FH and SDHB show a CpG island methylator phenotype (CIMP)
Source: PLoS One. 2022 Dec 1;17(12):e0278108. doi: 10.1371/journal.pone.0278108 (PMC9714951; doi:10.1371/journal.pone.0278108)
Supplement: S1 File — (DOCX) [file pone.0278108.s007.docx]

**S1 File. S1-S7 Figs**


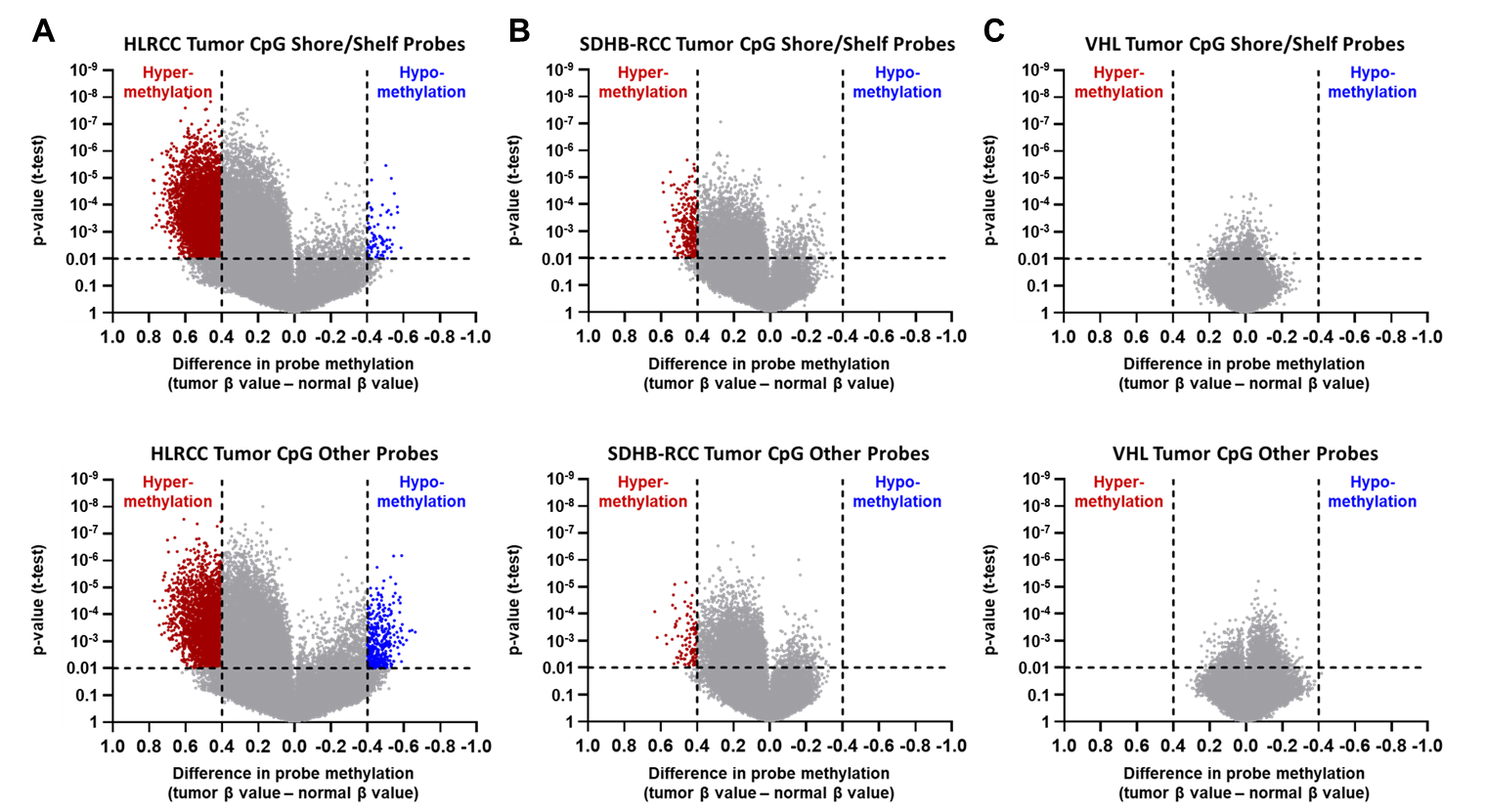


**S1 Fig. Volcano plots for non-CpG island probes.**

Volcano plots were used to evaluate either the CpG Shore/Shelf or CpG Other probe methylation status for the 4 tumor/normal samples pairs for HLRCC or SDHB-RCC patients and the 3 tumor/normal samples pairs for VHL patients. Probes with an average tumor difference in β-value of either 0.4 or -0.4 in comparison to the normal with a p-value of less than 0,01 were considered hyper- or hypomethylated respectively.


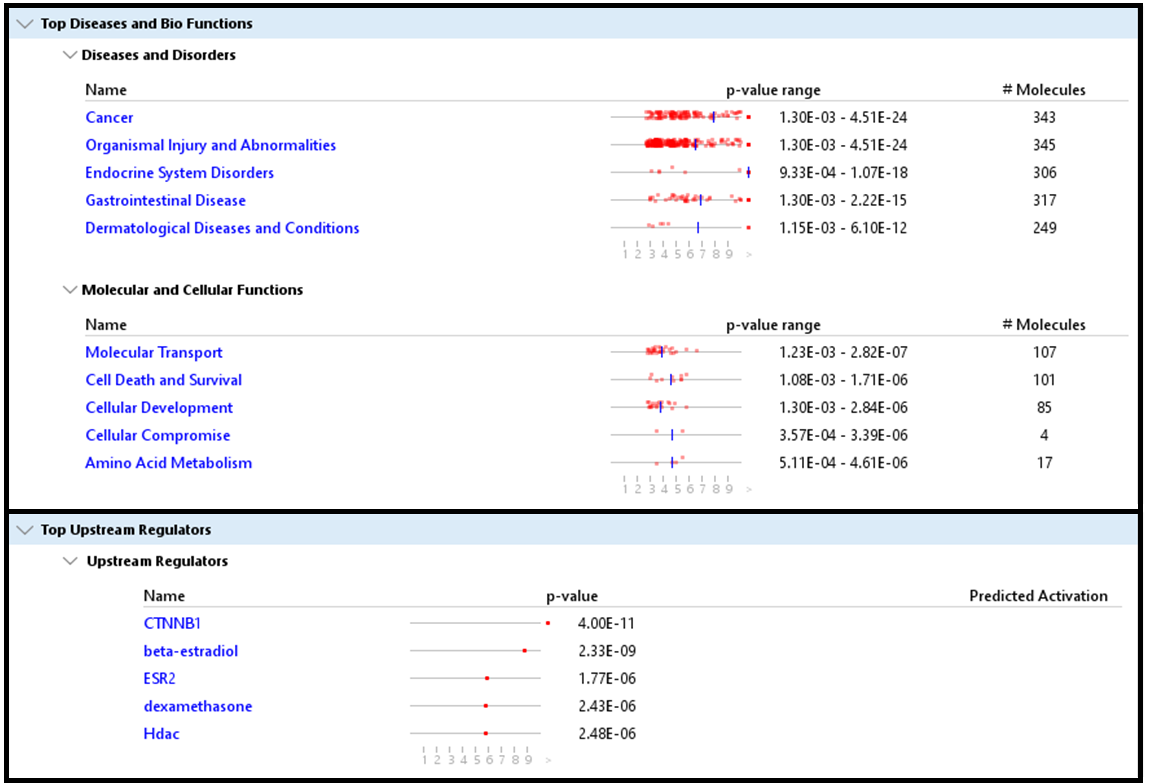


**S2 Fig. In-silico Ingenuity-based pathway analysis.**

Summary of the core analysis by IPA (<http://www.ingenuity.com>) of the 352 genes that are both hypermethylated and downregulated in HLRCC tumors. Notably, cancer was the highest associated disease and β-catenin (CTNNB1) was the highest associated upstream regulator.


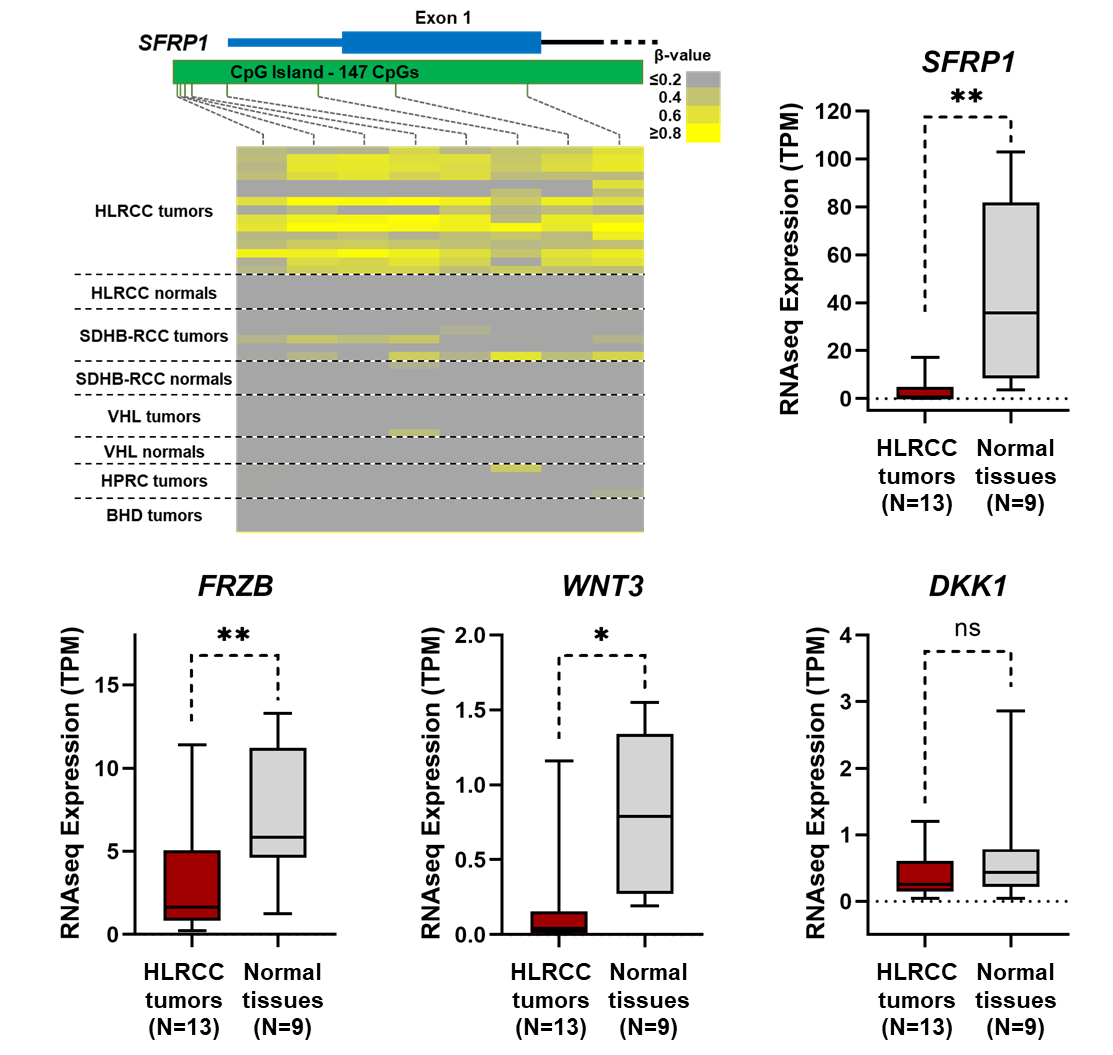


**S3 Fig. Hypermethylation of *SFRP1* in HLRCC tumors.**

A) Methylation β-value heatmaps for all the Illumina HumanMethylation450 BeadChip array probes within the CpG islands of the *SFRP1* gene. B) mRNA expression graphs for *SFRP1* comparing 13 HLRCC tumors with 9 normal tissues (5 from HLRCC patients and 4 from unaffected patients). D) mRNA expression graphs for the associated WNT pathway genes including *FRZB*, *WNT3*, and *DKK1*.


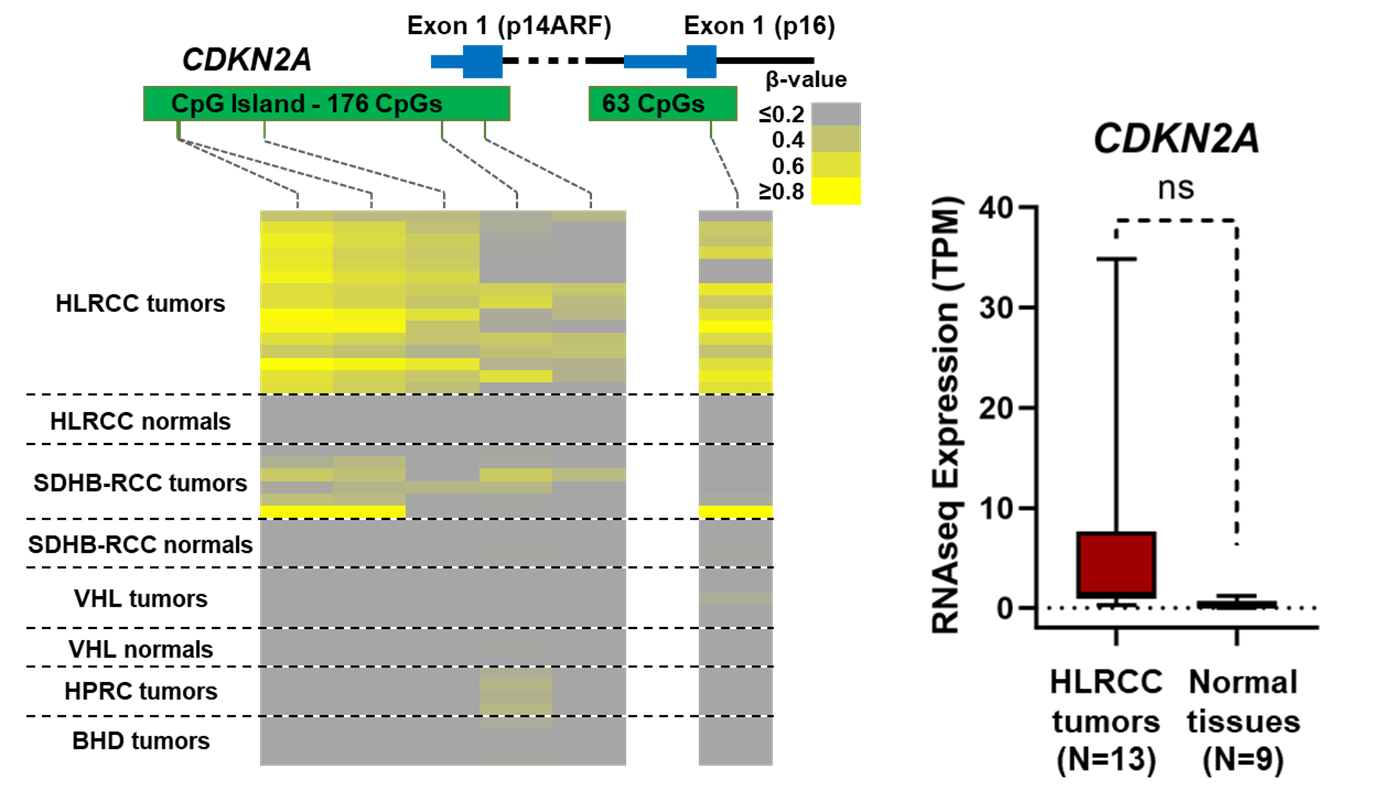


**S4 Fig. Hypermethylation of *CDKN2A*  in HLRCC tumors.**

Methylation β-value heatmaps for all the Illumina HumanMethylation450 BeadChip array probes within the CpG islands of the *SFRP1* gene and mRNA expression for *CDKN2A* comparing 13 HLRCC tumors with 9 normal tissues (5 from HLRCC patients and 4 from unaffected patients).


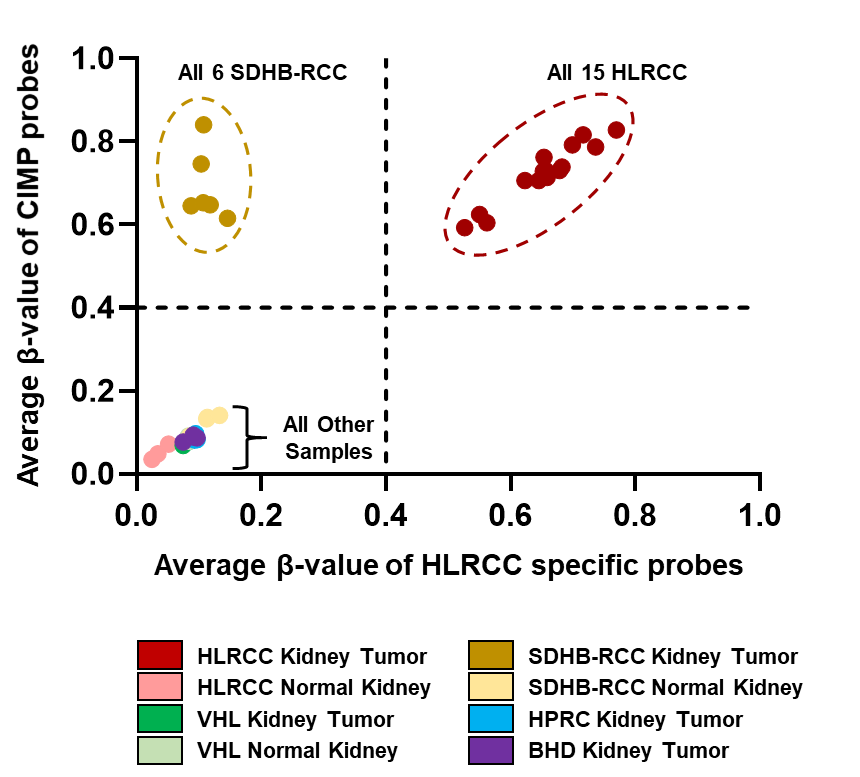


**S5 Fig. Selected 50-probe panel identifies CIMP tumors.**

A set of 25 CIMP probes and 25 HLRCC specific probes that were highly methylated for these two criteria were identified and average values for both probe sets were calculated for each tumor and graphed. This 50-probe panel could identify all the CIMP samples in the cohort and differentiate between HLRCC and SDHB-RCC tumors. This analysis used all available probes in the Illumina HumanMethylation450 BeadChip array.

**
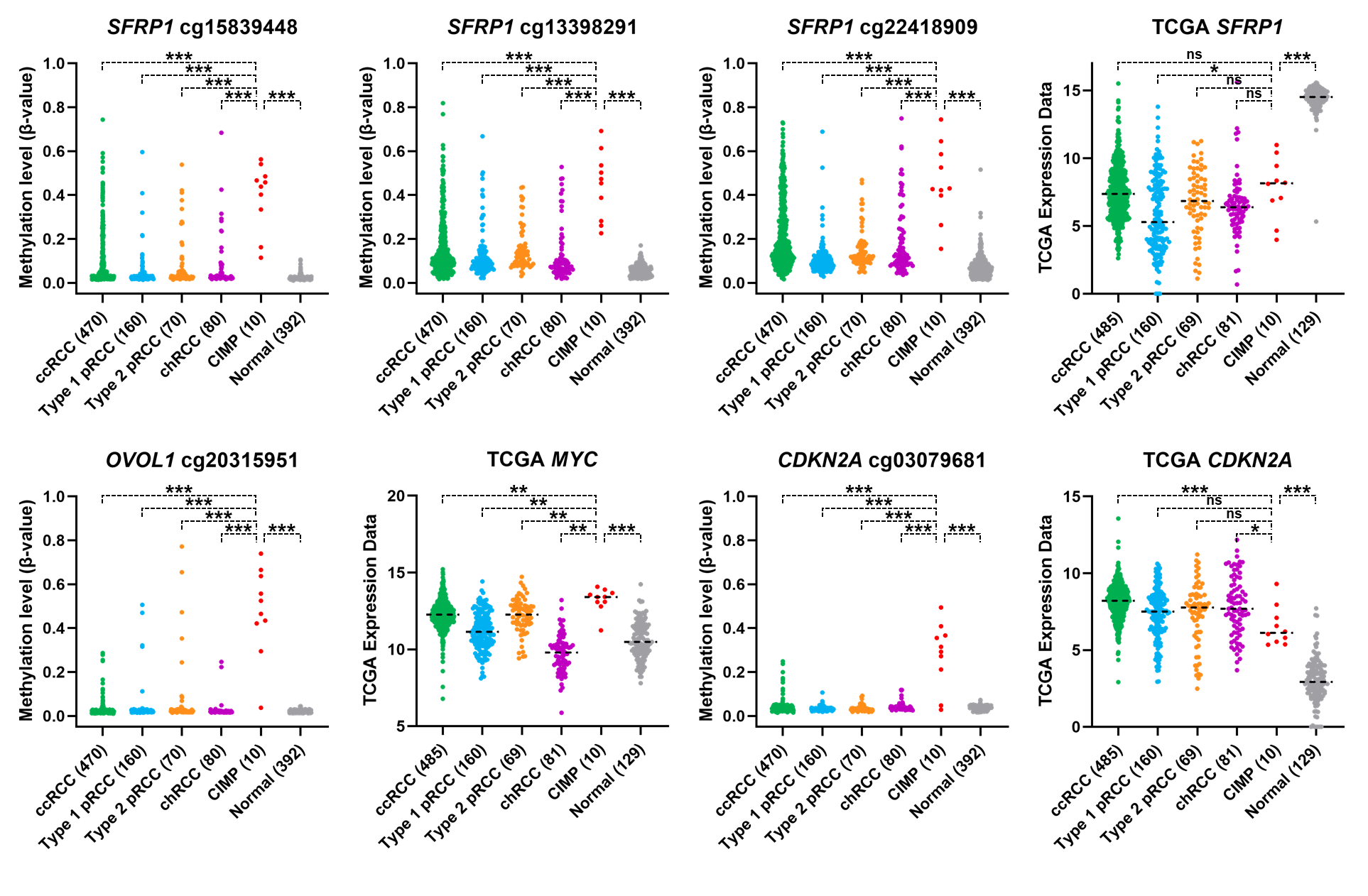
**

**S6 Fig. Confirmatory analysis of TCGA RCC tumors.**

Illumina HumanMethylation450 BeadChip array probes within the publicly available data from the Cancer Genome Atlas (TCGA) for the *SFRP1*, *OVOL1* and *CDKN2A* genes were identified. The methylation status of each probe was compared between the CIMP tumors identified within the TCGA cohort and the other major histologic RCC subtypes. This data was available for 790 RCC tumors and 392 associated normal kidney samples. The expression data from the TCGA RNAseq analysis of RCC for *SFRP1*, *MYC* (a downstream target of OVOL1), and *CDKN2A* was used to compare the relative expression profiles of the CIMP tumors and the other major histologic RCC subtypes. This data was available for 805 RCC tumors and 129 associated normal kidney samples.

All statistics were t-tests and p-values were defined as * <0.05, ** <0.001, *** <0.0001

**
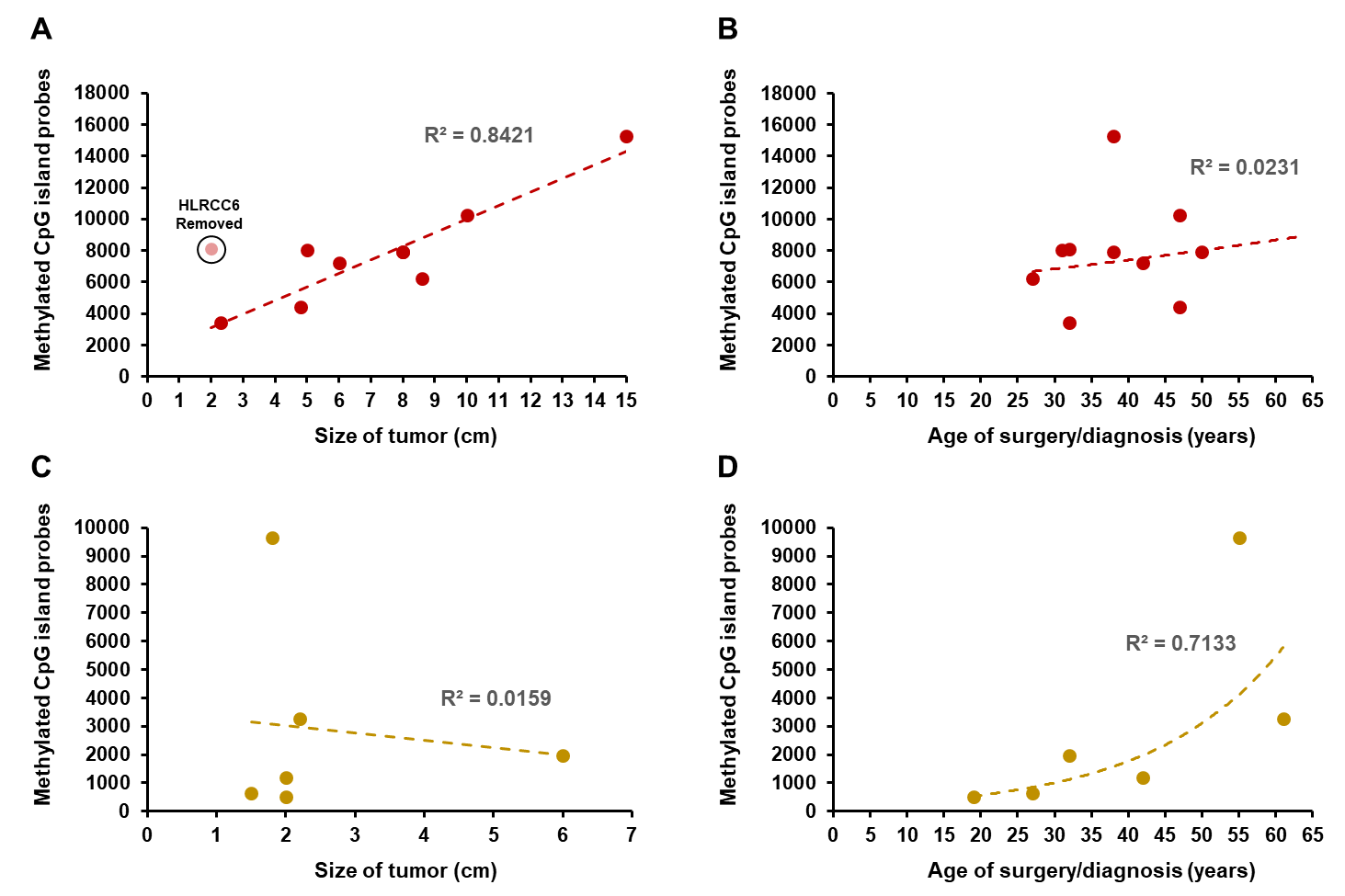
**

**S7 Fig. Correlation of hypermethylation level with size of tumor and age at diagnosis/surgery.**

The general level of hypermethylation within each tumor was calculated by first identifying all CpG island probes with β-values less than 0.2 in all 11 associated normal samples (4x HLRCC normal, 4x SDHB-RCC normal, and 3x VHL normal) were identified (n=31287). Then, for each HLRCC or SDHB-RCC primary kidney tumor the number of these probes where the β-value was greater than 0.5 was calculated and used as a representative level of hypermethylation.

A) Comparison of level of hypermethylation in HLRCC tumors with size of tumor, in this case an outlier, HLRCC6, was removed to show an improved correlation. B) Comparison of level of hypermethylation in HLRCC tumors with age of surgery/diagnosis. C) Comparison of level of hypermethylation in SDHB-RCC tumors with size of tumor. A) Comparison of level of hypermethylation in SDHB-RCC tumors with age of surgery/diagnosis.
